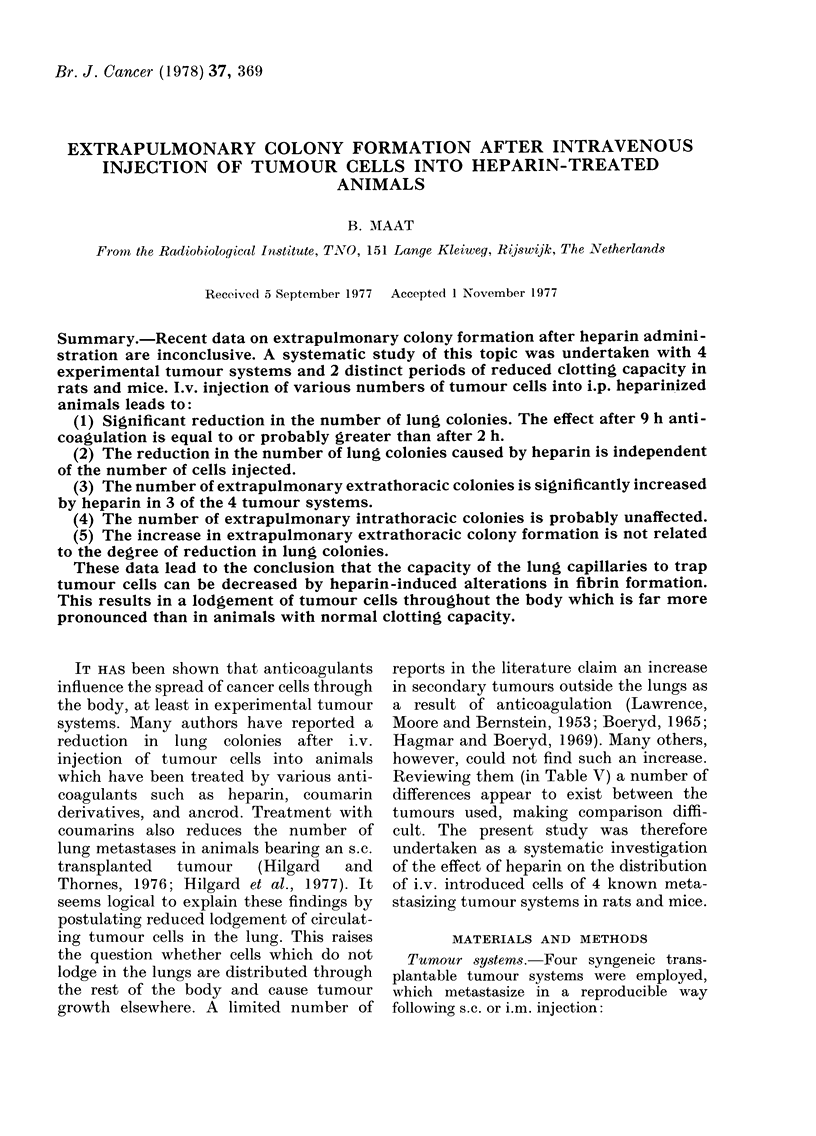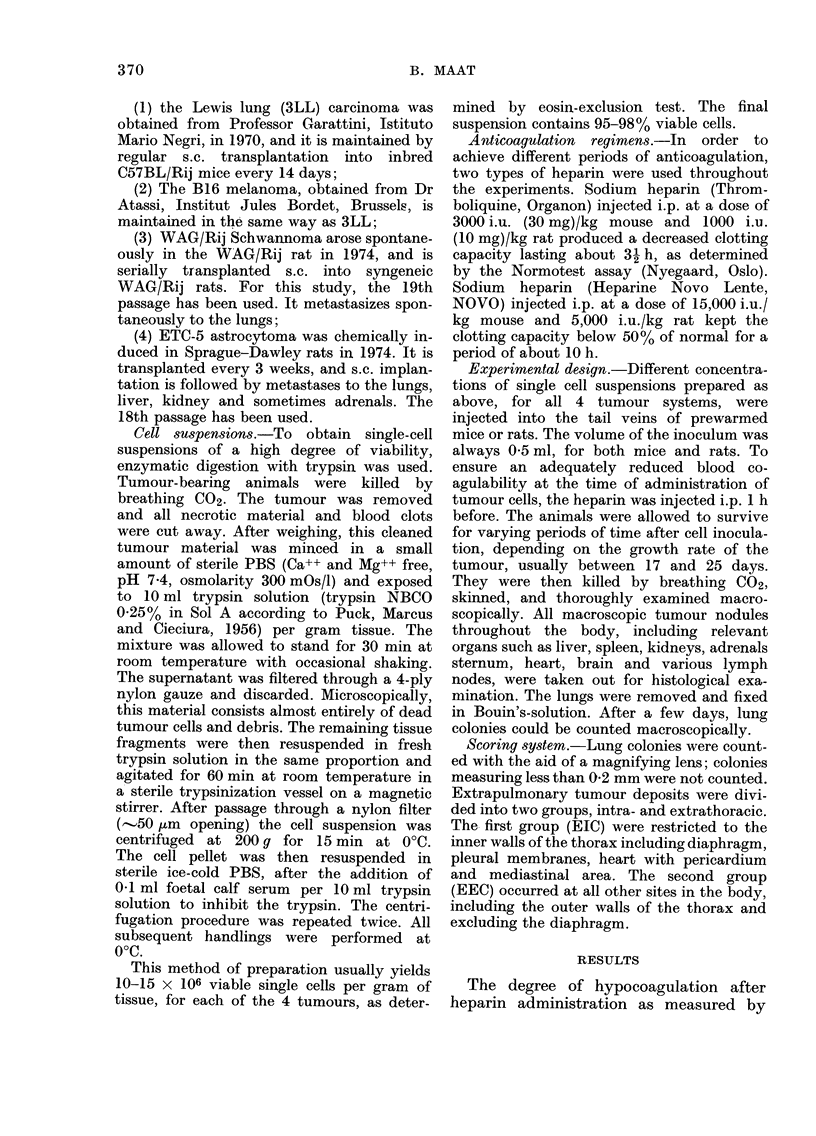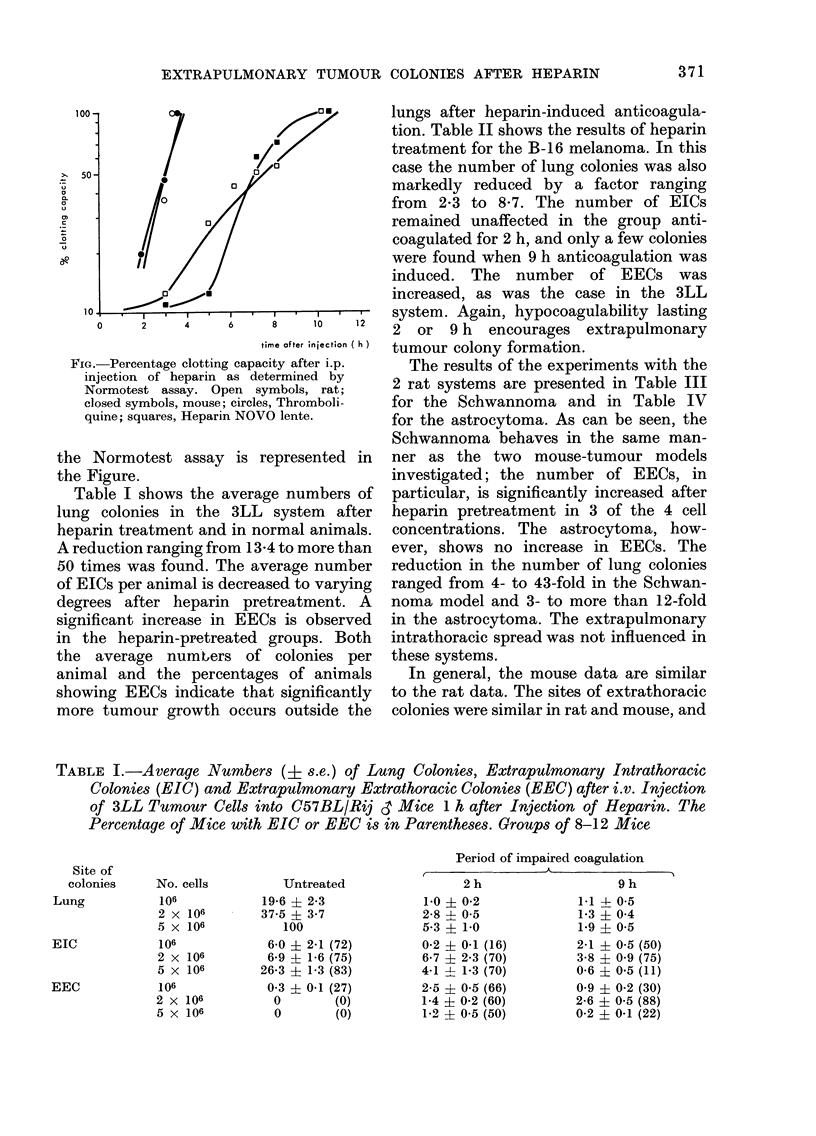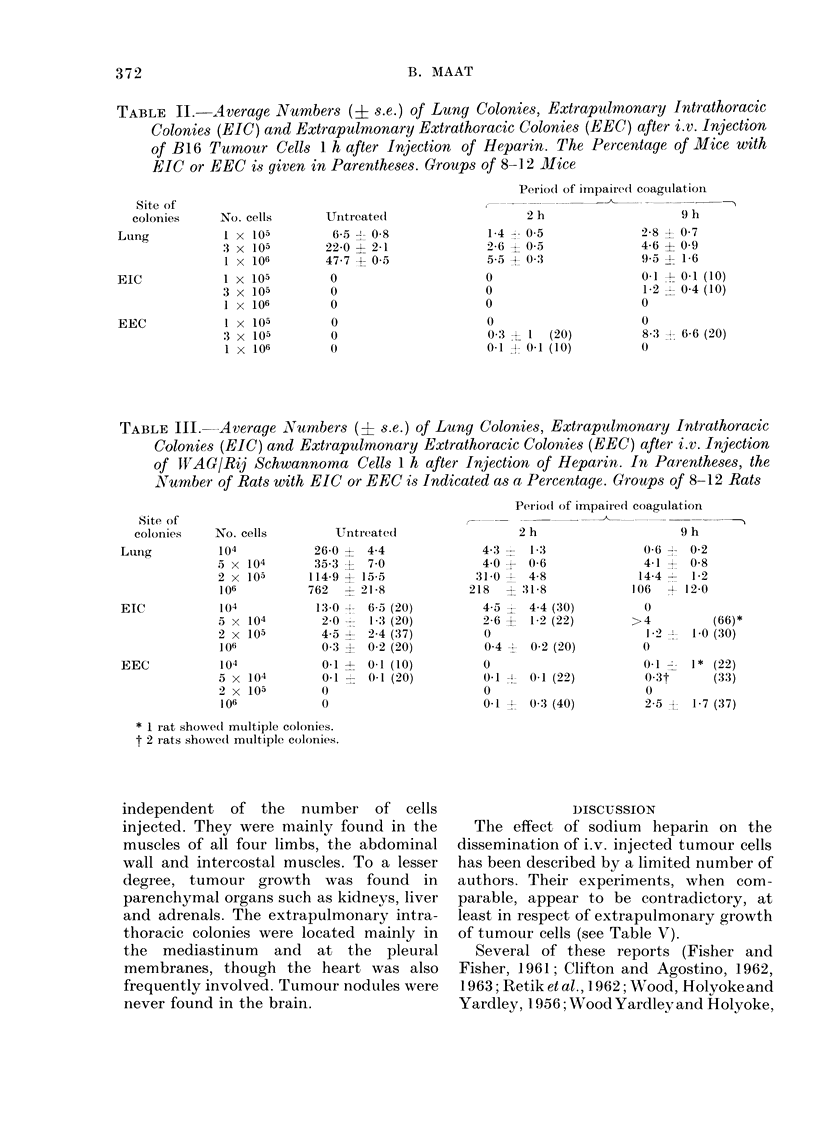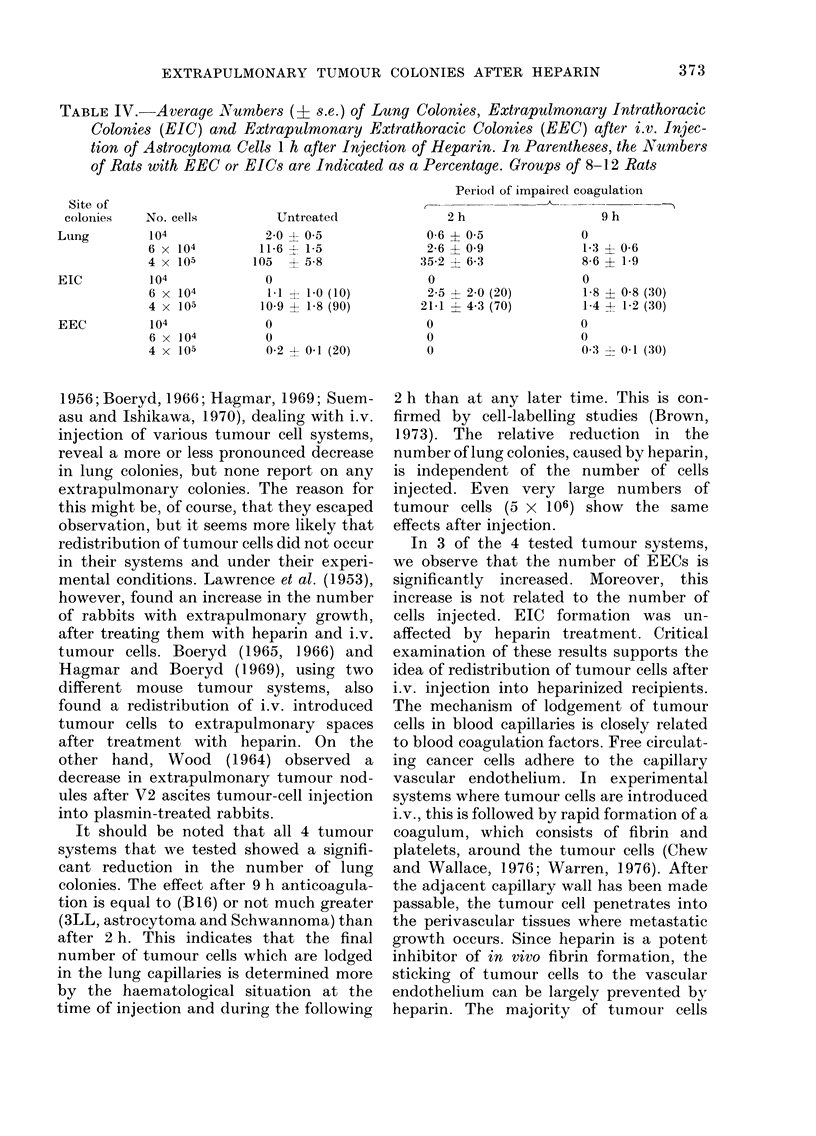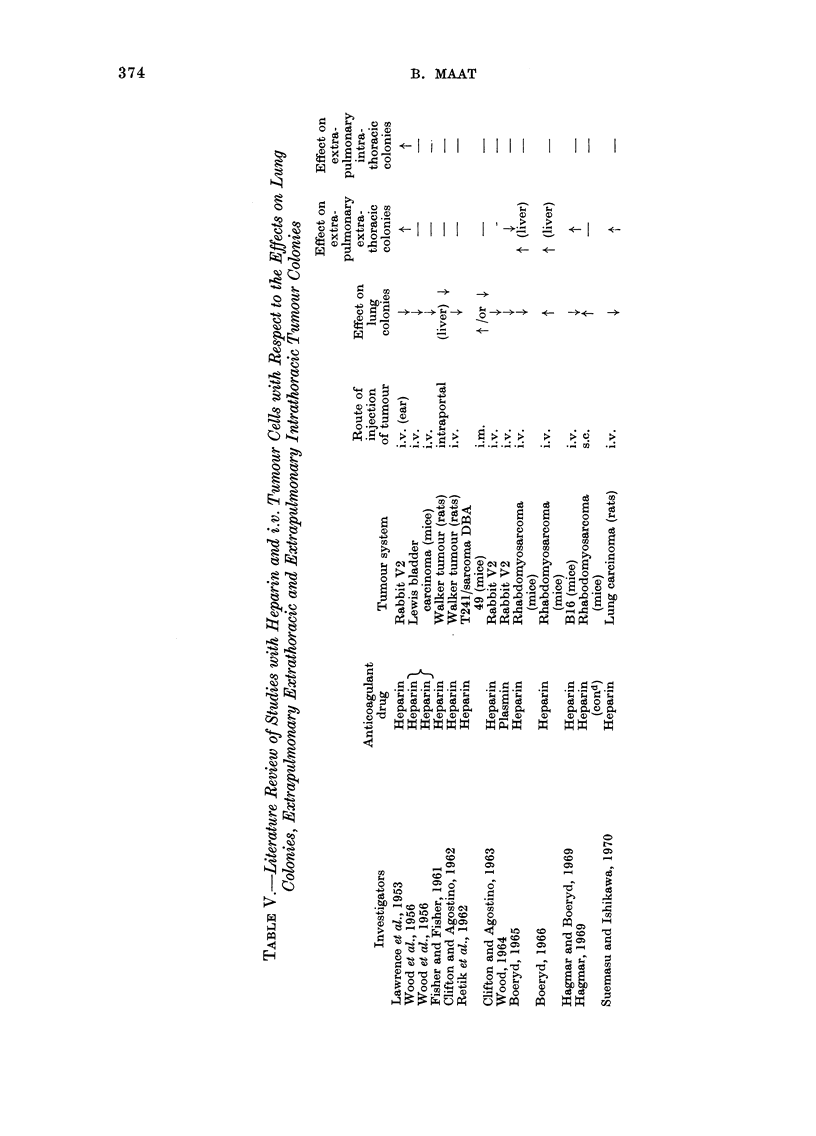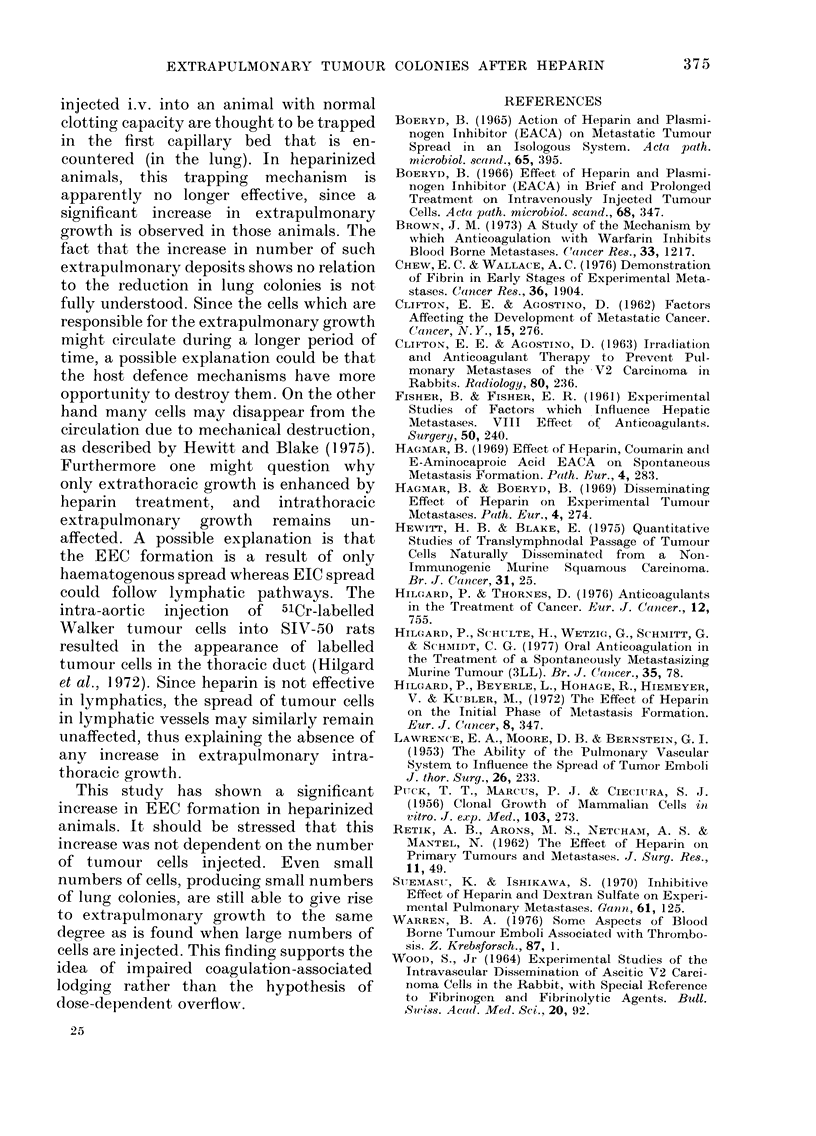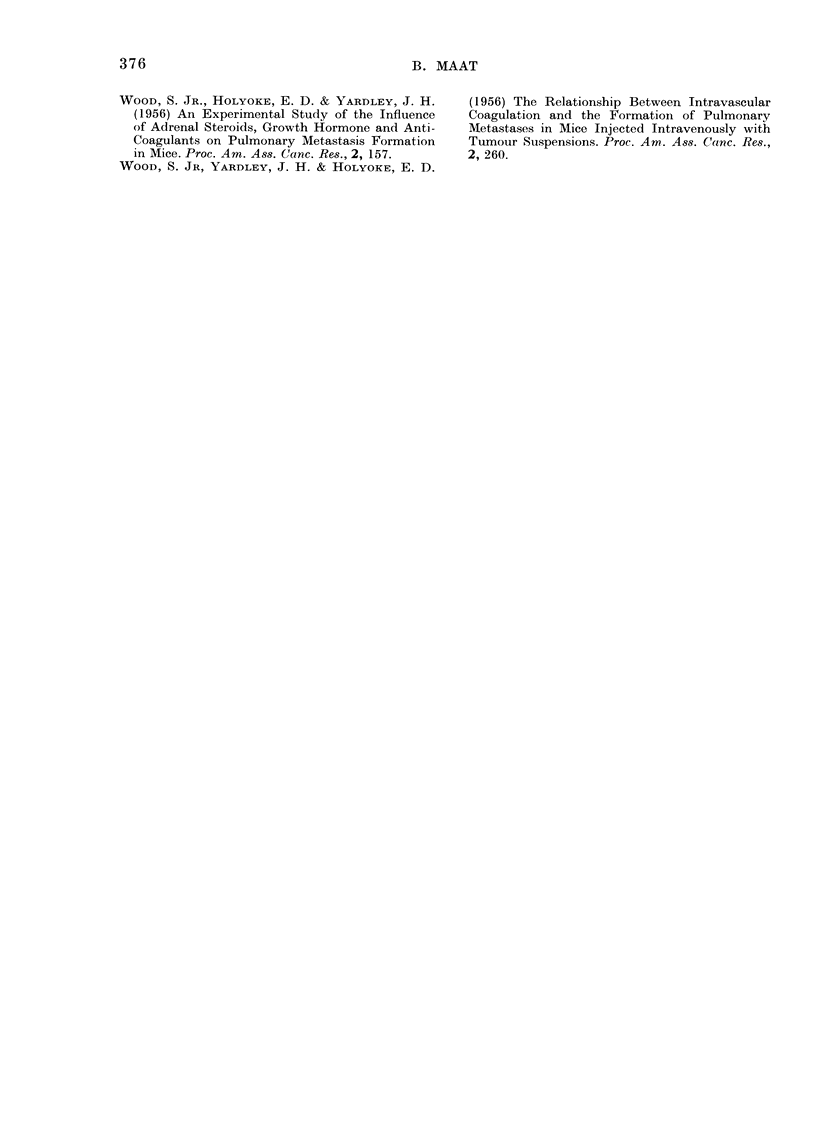# Extrapulmonary colony formation after intravenous injection of tumour cells into heparin-treated animals.

**DOI:** 10.1038/bjc.1978.56

**Published:** 1978-03

**Authors:** B. Maat

## Abstract

Recent data on extrapulmonary colony formation after heparin administration are inconclusive. A systematic study of this topic was undertaken with 4 experimental tumour systems and 2 distinct periods of reduced clotting capacity in rats and mice. I.v. injection of various numbers of tumour cells into i.p. heparinized animals leads to: (1) Significant reduction in the number of lung colonies. The effect after 9 h anti-coagulation is equal to or probably greater than after 2 h. (2) The reduction in the number of lung colonies caused by heparin is independent of the number of cells injected. (3) The number of extrapulmonary extrathoracic colonies is significantly increased by heparin in 3 of the 4 tumour systems. (4) The number of extrapulmonary intrathoracic colonies is probably unaffected. (5) The increase in extrapulmonary extrathoracic colony formation is not related to the degree of reduction in lung colonies. These data lead to the conclusion that the capacity of the lung capillaries to trap tumour cells can be decreased by heparin-induced alterations in fibrin formation. This results in a lodgement of tumour cells throughout the body which is far more pronounced than in animals with normal clotting capacity.


Br. J. Cancer (1978) 37, 369

### EXTRAPULMONARY COLONY FORMATION AFTER INTRAVENOUS INJECTION OF TUMOUR CELLS INTO HEPARIN-TREATED ANIMALS

B. MAAT

*From the Radiobiological Institute, TNO, 151 Lange Kleiweg, Rijswijk, The Netherlands*

Received 5 September 1977 Accepted 1 November 1977

**Summary.—Recent data on extrapulmonary colony formation after heparin administration are inconclusive. A systematic study of this topic was undertaken with 4 experimental tumour systems and 2 distinct periods of reduced clotting capacity in rats and mice. I.v. injection of various numbers of tumour cells into i.p. heparinized animals leads to:**

**(1) Significant reduction in the number of lung colonies. The effect after 9 h anticoagulation is equal to or probably greater than after 2 h.**

**(2) The reduction in the number of lung colonies caused by heparin is independent of the number of cells injected.**

**(3) The number of extrapulmonary extrathoracic colonies is significantly increased by heparin in 3 of the 4 tumour systems.**

**(4) The number of extrapulmonary intrathoracic colonies is probably unaffected.**

**(5) The increase in extrapulmonary extrathoracic colony formation is not related to the degree of reduction in lung colonies.**

**These data lead to the conclusion that the capacity of the lung capillaries to trap tumour cells can be decreased by heparin-induced alterations in fibrin formation. This results in a lodgement of tumour cells throughout the body which is far more pronounced than in animals with normal clotting capacity.**

It has been shown that anticoagulants influence the spread of cancer cells through the body, at least in experimental tumour systems. Many authors have reported a reduction in lung colonies after i.v. injection of tumour cells into animals which have been treated by various anticoagulants such as heparin, coumarin derivatives, and ancrod. Treatment with coumarins also reduces the number of lung metastases in animals bearing an s.c. transplanted tumour (Hilgard and Thornes, 1976; Hilgard *et al.*, 1977). It seems logical to explain these findings by postulating reduced lodgement of circulating tumour cells in the lung. This raises the question whether cells which do not lodge in the lungs are distributed through the rest of the body and cause tumour growth elsewhere. A limited number of reports in the literature claim an increase in secondary tumours outside the lungs as a result of anticoagulation (Lawrence, Moore and Bernstein, 1953; Boeryd, 1965; Hagmar and Boeryd, 1969). Many others, however, could not find such an increase. Reviewing them (in Table V) a number of differences appear to exist between the tumours used, making comparison difficult. The present study was therefore undertaken as a systematic investigation of the effect of heparin on the distribution of i.v. introduced cells of 4 known metastasizing tumour systems in rats and mice.

### MATERIALS AND METHODS

*Tumour systems.*—Four syngeneic transplantable tumour systems were employed, which metastasize in a reproducible way following s.c. or i.m. injection:

(1) the Lewis lung (3LL) carcinoma was obtained from Professor Garattini, Istituto Mario Negri, in 1970, and it is maintained by regular s.c. transplantation into inbred C57BL/Rij mice every 14 days;

(2) The B16 melanoma, obtained from Dr Atassi, Institut Jules Bordet, Brussels, is maintained in the same way as 3LL;

(3) WAG/Rij Schwannoma arose spontaneously in the WAG/Rij rat in 1974, and is serially transplanted s.c. into syngeneic WAG/Rij rats. For this study, the 19th passage has been used. It metastasizes spontaneously to the lungs;

(4) ETC-5 astrocytoma was chemically induced in Sprague–Dawley rats in 1974. It is transplanted every 3 weeks, and s.c. implantation is followed by metastases to the lungs, liver, kidney and sometimes adrenals. The 18th passage has been used.

*Cell suspensions.*—To obtain single-cell suspensions of a high degree of viability, enzymatic digestion with trypsin was used. Tumour-bearing animals were killed by breathing $CO_2$. The tumour was removed and all necrotic material and blood clots were cut away. After weighing, this cleaned tumour material was minced in a small amount of sterile PBS ($Ca^{++}$ and $Mg^{++}$ free, pH 7·4, osmolarity 300 mOs/l) and exposed to 10 ml trypsin solution (trypsin NBCO 0·25% in Sol A according to Puck, Marcus and Cieciura, 1956) per gram tissue. The mixture was allowed to stand for 30 min at room temperature with occasional shaking. The supernatant was filtered through a 4-ply nylon gauze and discarded. Microscopically, this material consists almost entirely of dead tumour cells and debris. The remaining tissue fragments were then resuspended in fresh trypsin solution in the same proportion and agitated for 60 min at room temperature in a sterile trypsinization vessel on a magnetic stirrer. After passage through a nylon filter ($\sim$50 $\mu$m opening) the cell suspension was centrifuged at 200 $g$ for 15 min at 0°C. The cell pellet was then resuspended in sterile ice-cold PBS, after the addition of 0·1 ml foetal calf serum per 10 ml trypsin solution to inhibit the trypsin. The centrifugation procedure was repeated twice. All subsequent handlings were performed at 0°C.

This method of preparation usually yields 10–15 × $10^6$ viable single cells per gram of tissue, for each of the 4 tumours, as determined by eosin-exclusion test. The final suspension contains 95–98% viable cells.

*Anticoagulation regimens.*—In order to achieve different periods of anticoagulation, two types of heparin were used throughout the experiments. Sodium heparin (Thromboliquine, Organon) injected i.p. at a dose of 3000 i.u. (30 mg)/kg mouse and 1000 i.u. (10 mg)/kg rat produced a decreased clotting capacity lasting about $3\frac{1}{2}$ h, as determined by the Normotest assay (Nyegaard, Oslo). Sodium heparin (Heparine Novo Lente, NOVO) injected i.p. at a dose of 15,000 i.u./kg mouse and 5,000 i.u./kg rat kept the clotting capacity below 50% of normal for a period of about 10 h.

*Experimental design.*—Different concentrations of single cell suspensions prepared as above, for all 4 tumour systems, were injected into the tail veins of prewarmed mice or rats. The volume of the inoculum was always 0·5 ml, for both mice and rats. To ensure an adequately reduced blood coagulability at the time of administration of tumour cells, the heparin was injected i.p. 1 h before. The animals were allowed to survive for varying periods of time after cell inoculation, depending on the growth rate of the tumour, usually between 17 and 25 days. They were then killed by breathing $CO_2$, skinned, and thoroughly examined macroscopically. All macroscopic tumour nodules throughout the body, including relevant organs such as liver, spleen, kidneys, adrenals sternum, heart, brain and various lymph nodes, were taken out for histological examination. The lungs were removed and fixed in Bouin's-solution. After a few days, lung colonies could be counted macroscopically.

*Scoring system.*—Lung colonies were counted with the aid of a magnifying lens; colonies measuring less than 0·2 mm were not counted. Extrapulmonary tumour deposits were divided into two groups, intra- and extrathoracic. The first group (EIC) were restricted to the inner walls of the thorax including diaphragm, pleural membranes, heart with pericardium and mediastinal area. The second group (EEC) occurred at all other sites in the body, including the outer walls of the thorax and excluding the diaphragm.

### RESULTS

The degree of hypocoagulation after heparin administration as measured by

FIG.—Percentage clotting capacity after i.p. injection of heparin as determined by Normotest assay. Open symbols, rat; closed symbols, mouse; circles, Thromboliquine; squares, Heparin NOVO lente.

the Normotest assay is represented in the Figure.

Table I shows the average numbers of lung colonies in the 3LL system after heparin treatment and in normal animals. A reduction ranging from 13·4 to more than 50 times was found. The average number of EICs per animal is decreased to varying degrees after heparin pretreatment. A significant increase in EECs is observed in the heparin-pretreated groups. Both the average numbers of colonies per animal and the percentages of animals showing EECs indicate that significantly more tumour growth occurs outside the lungs after heparin-induced anticoagulation. Table II shows the results of heparin treatment for the B-16 melanoma. In this case the number of lung colonies was also markedly reduced by a factor ranging from 2·3 to 8·7. The number of EICs remained unaffected in the group anticoagulated for 2 h, and only a few colonies were found when 9 h anticoagulation was induced. The number of EECs was increased, as was the case in the 3LL system. Again, hypocoagulability lasting 2 or 9 h encourages extrapulmonary tumour colony formation.

The results of the experiments with the 2 rat systems are presented in Table III for the Schwannoma and in Table IV for the astrocytoma. As can be seen, the Schwannoma behaves in the same manner as the two mouse-tumour models investigated; the number of EECs, in particular, is significantly increased after heparin pretreatment in 3 of the 4 cell concentrations. The astrocytoma, however, shows no increase in EECs. The reduction in the number of lung colonies ranged from 4- to 43-fold in the Schwannoma model and 3- to more than 12-fold in the astrocytoma. The extrapulmonary intrathoracic spread was not influenced in these systems.

In general, the mouse data are similar to the rat data. The sites of extrathoracic colonies were similar in rat and mouse, and

TABLE I.—*Average Numbers (± s.e.) of Lung Colonies, Extrapulmonary Intrathoracic Colonies (EIC) and Extrapulmonary Extrathoracic Colonies (EEC) after i.v. Injection of 3LL Tumour Cells into C57BL/Rij ♂ Mice 1 h after Injection of Heparin. The Percentage of Mice with EIC or EEC is in Parentheses. Groups of 8–12 Mice*

| Site of colonies | No. cells | Untreated | Period of impaired coagulation 2 h | 9 h |
|---|---|---|---|---|
| Lung | $10^6$ | 19·6 ± 2·3 | 1·0 ± 0·2 | 1·1 ± 0·5 |
| | $2 \times 10^6$ | 37·5 ± 3·7 | 2·8 ± 0·5 | 1·3 ± 0·4 |
| | $5 \times 10^6$ | 100 | 5·3 ± 1·0 | 1·9 ± 0·5 |
| EIC | $10^6$ | 6·0 ± 2·1 (72) | 0·2 ± 0·1 (16) | 2·1 ± 0·5 (50) |
| | $2 \times 10^6$ | 6·9 ± 1·6 (75) | 6·7 ± 2·3 (70) | 3·8 ± 0·9 (75) |
| | $5 \times 10^6$ | 26·3 ± 1·3 (83) | 4·1 ± 1·3 (70) | 0·6 ± 0·5 (11) |
| EEC | $10^6$ | 0·3 ± 0·1 (27) | 2·5 ± 0·5 (66) | 0·9 ± 0·2 (30) |
| | $2 \times 10^6$ | 0 (0) | 1·4 ± 0·2 (60) | 2·6 ± 0·5 (88) |
| | $5 \times 10^6$ | 0 (0) | 1·2 ± 0·5 (50) | 0·2 ± 0·1 (22) |

TABLE II.—*Average Numbers* ($\pm$ *s.e.*) *of Lung Colonies, Extrapulmonary Intrathoracic Colonies* (*EIC*) *and Extrapulmonary Extrathoracic Colonies* (*EEC*) *after i.v. Injection of B*16 *Tumour Cells* 1 *h after Injection of Heparin. The Percentage of Mice with EIC or EEC is given in Parentheses. Groups of* 8–12 *Mice*

| Site of colonies | No. cells | Untreated | Period of impaired coagulation: 2 h | Period of impaired coagulation: 9 h |
|---|---|---|---|---|
| Lung | $1 \times 10^5$ | 6·5 ± 0·8 | 1·4 ± 0·5 | 2·8 ± 0·7 |
| | $3 \times 10^5$ | 22·0 ± 2·1 | 2·6 ± 0·5 | 4·6 ± 0·9 |
| | $1 \times 10^6$ | 47·7 ± 0·5 | 5·5 ± 0·3 | 9·5 ± 1·6 |
| EIC | $1 \times 10^5$ | 0 | 0 | 0·1 ± 0·1 (10) |
| | $3 \times 10^5$ | 0 | 0 | 1·2 ± 0·4 (10) |
| | $1 \times 10^6$ | 0 | 0 | 0 |
| EEC | $1 \times 10^5$ | 0 | 0 | 0 |
| | $3 \times 10^5$ | 0 | 0·3 ± 1 (20) | 8·3 ± 6·6 (20) |
| | $1 \times 10^6$ | 0 | 0·1 ± 0·1 (10) | 0 |

TABLE III.—*Average Numbers* ($\pm$ *s.e.*) *of Lung Colonies, Extrapulmonary Intrathoracic Colonies* (*EIC*) *and Extrapulmonary Extrathoracic Colonies* (*EEC*) *after i.v. Injection of WAG/Rij Schwannoma Cells* 1 *h after Injection of Heparin. In Parentheses, the Number of Rats with EIC or EEC is Indicated as a Percentage. Groups of* 8–12 *Rats*

| Site of colonies | No. cells | Untreated | Period of impaired coagulation: 2 h | Period of impaired coagulation: 9 h |
|---|---|---|---|---|
| Lung | $10^4$ | 26·0 ± 4·4 | 4·3 ± 1·3 | 0·6 ± 0·2 |
| | $5 \times 10^4$ | 35·3 ± 7·0 | 4·0 ± 0·6 | 4·1 ± 0·8 |
| | $2 \times 10^5$ | 114·9 ± 15·5 | 31·0 ± 4·8 | 14·4 ± 1·2 |
| | $10^6$ | 762 ± 21·8 | 218 ± 31·8 | 106 ± 12·0 |
| EIC | $10^4$ | 13·0 ± 6·5 (20) | 4·5 ± 4·4 (30) | 0 |
| | $5 \times 10^4$ | 2·0 ± 1·3 (20) | 2·6 ± 1·2 (22) | >4 (66)* |
| | $2 \times 10^5$ | 4·5 ± 2·4 (37) | 0 | 1·2 ± 1·0 (30) |
| | $10^6$ | 0·3 ± 0·2 (20) | 0·4 ± 0·2 (20) | 0 |
| EEC | $10^4$ | 0·1 ± 0·1 (10) | 0 | 0·1 ± 1* (22) |
| | $5 \times 10^4$ | 0·1 ± 0·1 (20) | 0·1 ± 0·1 (22) | 0·3† (33) |
| | $2 \times 10^5$ | 0 | 0 | 0 |
| | $10^6$ | 0 | 0·1 ± 0·3 (40) | 2·5 ± 1·7 (37) |

* 1 rat showed multiple colonies.
† 2 rats showed multiple colonies.

independent of the number of cells injected. They were mainly found in the muscles of all four limbs, the abdominal wall and intercostal muscles. To a lesser degree, tumour growth was found in parenchymal organs such as kidneys, liver and adrenals. The extrapulmonary intrathoracic colonies were located mainly in the mediastinum and at the pleural membranes, though the heart was also frequently involved. Tumour nodules were never found in the brain.

### DISCUSSION

The effect of sodium heparin on the dissemination of i.v. injected tumour cells has been described by a limited number of authors. Their experiments, when comparable, appear to be contradictory, at least in respect of extrapulmonary growth of tumour cells (see Table V).

Several of these reports (Fisher and Fisher, 1961; Clifton and Agostino, 1962, 1963; Retik *et al.*, 1962; Wood, Holyoke and Yardley, 1956; Wood Yardley and Holyoke,

TABLE IV.—*Average Numbers* (± *s.e.*) *of Lung Colonies, Extrapulmonary Intrathoracic Colonies* (*EIC*) *and Extrapulmonary Extrathoracic Colonies* (*EEC*) *after i.v. Injection of Astrocytoma Cells* 1 *h after Injection of Heparin. In Parentheses, the Numbers of Rats with EEC or EICs are Indicated as a Percentage. Groups of* 8–12 *Rats*

| Site of colonies | No. cells | Untreated | Period of impaired coagulation 2 h | 9 h |
|---|---|---|---|---|
| Lung | $10^4$ | 2·0 ± 0·5 | 0·6 ± 0·5 | 0 |
| | $6 \times 10^4$ | 11·6 ± 1·5 | 2·6 ± 0·9 | 1·3 ± 0·6 |
| | $4 \times 10^5$ | 105 ± 5·8 | 35·2 ± 6·3 | 8·6 ± 1·9 |
| EIC | $10^4$ | 0 | 0 | 0 |
| | $6 \times 10^4$ | 1·1 ± 1·0 (10) | 2·5 ± 2·0 (20) | 1·8 ± 0·8 (30) |
| | $4 \times 10^5$ | 10·9 ± 1·8 (90) | 21·1 ± 4·3 (70) | 1·4 ± 1·2 (30) |
| EEC | $10^4$ | 0 | 0 | 0 |
| | $6 \times 10^4$ | 0 | 0 | 0 |
| | $4 \times 10^5$ | 0·2 ± 0·1 (20) | 0 | 0·3 ± 0·1 (30) |

1956; Boeryd, 1966; Hagmar, 1969; Suemasu and Ishikawa, 1970), dealing with i.v. injection of various tumour cell systems, reveal a more or less pronounced decrease in lung colonies, but none report on any extrapulmonary colonies. The reason for this might be, of course, that they escaped observation, but it seems more likely that redistribution of tumour cells did not occur in their systems and under their experimental conditions. Lawrence *et al.* (1953), however, found an increase in the number of rabbits with extrapulmonary growth, after treating them with heparin and i.v. tumour cells. Boeryd (1965, 1966) and Hagmar and Boeryd (1969), using two different mouse tumour systems, also found a redistribution of i.v. introduced tumour cells to extrapulmonary spaces after treatment with heparin. On the other hand, Wood (1964) observed a decrease in extrapulmonary tumour nodules after V2 ascites tumour-cell injection into plasmin-treated rabbits.

It should be noted that all 4 tumour systems that we tested showed a significant reduction in the number of lung colonies. The effect after 9 h anticoagulation is equal to (B16) or not much greater (3LL, astrocytoma and Schwannoma) than after 2 h. This indicates that the final number of tumour cells which are lodged in the lung capillaries is determined more by the haematological situation at the time of injection and during the following 2 h than at any later time. This is confirmed by cell-labelling studies (Brown, 1973). The relative reduction in the number of lung colonies, caused by heparin, is independent of the number of cells injected. Even very large numbers of tumour cells ($5 \times 10^6$) show the same effects after injection.

In 3 of the 4 tested tumour systems, we observe that the number of EECs is significantly increased. Moreover, this increase is not related to the number of cells injected. EIC formation was unaffected by heparin treatment. Critical examination of these results supports the idea of redistribution of tumour cells after i.v. injection into heparinized recipients. The mechanism of lodgement of tumour cells in blood capillaries is closely related to blood coagulation factors. Free circulating cancer cells adhere to the capillary vascular endothelium. In experimental systems where tumour cells are introduced i.v., this is followed by rapid formation of a coagulum, which consists of fibrin and platelets, around the tumour cells (Chew and Wallace, 1976; Warren, 1976). After the adjacent capillary wall has been made passable, the tumour cell penetrates into the perivascular tissues where metastatic growth occurs. Since heparin is a potent inhibitor of *in vivo* fibrin formation, the sticking of tumour cells to the vascular endothelium can be largely prevented by heparin. The majority of tumour cells

TABLE V.—*Literature Review of Studies with Heparin and i.v. Tumour Cells with Respect to the Effects on Lung Colonies, Extrapulmonary Extrathoracic and Extrapulmonary Intrathoracic Tumour Colonies*

| Investigators | Anticoagulant drug | Tumour system | Route of injection of tumour | Effect on lung colonies | Effect on extra-pulmonary extra-thoracic colonies | Effect on extra-pulmonary intra-thoracic colonies |
|---|---|---|---|---|---|---|
| Lawrence *et al.*, 1953 | Heparin | Rabbit V2 | i.v. (ear) | ↓ | ↑ | ↑ |
| Wood *et al.*, 1956 | Heparin } | Lewis bladder carcinoma (mice) | i.v. | ↓ | — | — |
| Wood *et al.*, 1956 | Heparin } | | i.v. | ↓ | — | — |
| Fisher and Fisher, 1961 | Heparin | Walker tumour (rats) | intraportal | (liver) ↓ | — | — |
| Clifton and Agostino, 1962 | Heparin | Walker tumour (rats) | i.v. | ↓ | — | — |
| Retik *et al.*, 1962 | Heparin | T241/sarcoma DBA 49 (mice) | i.m. | ↑ /or ↓ | — | — |
| Clifton and Agostino, 1963 | Heparin | Rabbit V2 | i.v. | ↓ | - | — |
| Wood, 1964 | Plasmin | Rabbit V2 | i.v. | ↓ | ↓ | — |
| Boeryd, 1965 | Heparin | Rhabdomyosarcoma (mice) | i.v. | ↓ | ↑ (liver) | — |
| Boeryd, 1966 | Heparin | Rhabdomyosarcoma (mice) | i.v. | ↑ | ↑ (liver) | — |
| Hagmar and Boeryd, 1969 | Heparin | B16 (mice) | i.v. | ↓ | ↑ | — |
| Hagmar, 1969 | Heparin (con[d]) | Rhabodomyosarcoma (mice) | s.c. | ↑ | — | — |
| Suemasu and Ishikawa, 1970 | Heparin | Lung carcinoma (rats) | i.v. | ↓ | ↑ | — |

injected i.v. into an animal with normal clotting capacity are thought to be trapped in the first capillary bed that is encountered (in the lung). In heparinized animals, this trapping mechanism is apparently no longer effective, since a significant increase in extrapulmonary growth is observed in those animals. The fact that the increase in number of such extrapulmonary deposits shows no relation to the reduction in lung colonies is not fully understood. Since the cells which are responsible for the extrapulmonary growth might circulate during a longer period of time, a possible explanation could be that the host defence mechanisms have more opportunity to destroy them. On the other hand many cells may disappear from the circulation due to mechanical destruction, as described by Hewitt and Blake (1975). Furthermore one might question why only extrathoracic growth is enhanced by heparin treatment, and intrathoracic extrapulmonary growth remains unaffected. A possible explanation is that the EEC formation is a result of only haematogenous spread whereas EIC spread could follow lymphatic pathways. The intra-aortic injection of $^{51}$Cr-labelled Walker tumour cells into SIV-50 rats resulted in the appearance of labelled tumour cells in the thoracic duct (Hilgard *et al.*, 1972). Since heparin is not effective in lymphatics, the spread of tumour cells in lymphatic vessels may similarly remain unaffected, thus explaining the absence of any increase in extrapulmonary intrathoracic growth.

This study has shown a significant increase in EEC formation in heparinized animals. It should be stressed that this increase was not dependent on the number of tumour cells injected. Even small numbers of cells, producing small numbers of lung colonies, are still able to give rise to extrapulmonary growth to the same degree as is found when large numbers of cells are injected. This finding supports the idea of impaired coagulation-associated lodging rather than the hypothesis of dose-dependent overflow.

### REFERENCES

BOERYD, B. (1965) Action of Heparin and Plasminogen Inhibitor (EACA) on Metastatic Tumour Spread in an Isologous System. *Acta path. microbiol. scand.*, **65**, 395.

BOERYD, B. (1966) Effect of Heparin and Plasminogen Inhibitor (EACA) in Brief and Prolonged Treatment on Intravenously Injected Tumour Cells. *Acta path. microbiol. scand.*, **68**, 347.

BROWN, J. M. (1973) A Study of the Mechanism by which Anticoagulation with Warfarin Inhibits Blood Borne Metastases. *Cancer Res.*, **33**, 1217.

CHEW, E. C. & WALLACE, A. C. (1976) Demonstration of Fibrin in Early Stages of Experimental Metastases. *Cancer Res.*, **36**, 1904.

CLIFTON, E. E. & AGOSTINO, D. (1962) Factors Affecting the Development of Metastatic Cancer. *Cancer, N.Y.*, **15**, 276.

CLIFTON, E. E. & AGOSTINO, D. (1963) Irradiation and Anticoagulant Therapy to Prevent Pulmonary Metastases of the V2 Carcinoma in Rabbits. *Radiology*, **80**, 236.

FISHER, B. & FISHER, E. R. (1961) Experimental Studies of Factors which Influence Hepatic Metastases. VIII Effect of Anticoagulants. *Surgery*, **50**, 240.

HAGMAR, B. (1969) Effect of Heparin, Coumarin and E-Aminocaproic Acid EACA on Spontaneous Metastasis Formation. *Path. Eur.*, **4**, 283.

HAGMAR, B. & BOERYD, B. (1969) Disseminating Effect of Heparin on Experimental Tumour Metastases. *Path. Eur.*, **4**, 274.

HEWITT, H. B. & BLAKE, E. (1975) Quantitative Studies of Translymphnodal Passage of Tumour Cells Naturally Disseminated from a Non-Immunogenic Murine Squamous Carcinoma. *Br. J. Cancer*, **31**, 25.

HILGARD, P. & THORNES, D. (1976) Anticoagulants in the Treatment of Cancer. *Eur. J. Cancer.*, **12**, 755.

HILGARD, P., SCHULTE, H., WETZIG, G., SCHMITT, G. & SCHMIDT, C. G. (1977) Oral Anticoagulation in the Treatment of a Spontaneously Metastasizing Murine Tumour (3LL). *Br. J. Cancer.*, **35**, 78.

HILGARD, P., BEYERLE, L., HOHAGE, R., HIEMEYER, V. & KUBLER, M., (1972) The Effect of Heparin on the Initial Phase of Metastasis Formation. *Eur. J. Cancer*, **8**, 347.

LAWRENCE, E. A., MOORE, D. B. & BERNSTEIN, G. I. (1953) The Ability of the Pulmonary Vascular System to Influence the Spread of Tumor Emboli *J. thor. Surg.*, **26**, 233.

PUCK, T. T., MARCUS, P. J. & CIECIURA, S. J. (1956) Clonal Growth of Mammalian Cells *in vitro*. *J. exp. Med.*, **103**, 273.

RETIK, A. B., ARONS, M. S., NETCHAM, A. S. & MANTEL, N. (1962) The Effect of Heparin on Primary Tumours and Metastases. *J. Surg. Res.*, **11**, 49.

SUEMASU, K. & ISHIKAWA, S. (1970) Inhibitive Effect of Heparin and Dextran Sulfate on Experimental Pulmonary Metastases. *Gann*, **61**, 125.

WARREN, B. A. (1976) Some Aspects of Blood Borne Tumour Emboli Associated with Thrombosis. *Z. Krebsforsch.*, **87**, 1.

WOOD, S., Jr (1964) Experimental Studies of the Intravascular Dissemination of Ascitic V2 Carcinoma Cells in the Rabbit, with Special Reference to Fibrinogen and Fibrinolytic Agents. *Bull. Swiss. Acad. Med. Sci.*, **20**, 92.

WOOD, S. JR., HOLYOKE, E. D. & YARDLEY, J. H. (1956) An Experimental Study of the Influence of Adrenal Steroids, Growth Hormone and Anti-Coagulants on Pulmonary Metastasis Formation in Mice. *Proc. Am. Ass. Canc. Res.*, **2**, 157.

WOOD, S. JR, YARDLEY, J. H. & HOLYOKE, E. D. (1956) The Relationship Between Intravascular Coagulation and the Formation of Pulmonary Metastases in Mice Injected Intravenously with Tumour Suspensions. *Proc. Am. Ass. Canc. Res.*, **2**, 260.